# Surveillance de l’efficacité des associations artémether-luméfantrine et artésunate/sulfadoxine-pyriméthamine dans le traitement du paludisme simple à *Plasmodium falciparum* à Boali, République centrafricaine

**DOI:** 10.48327/mtsi.v6i3.2026.883

**Published:** 2026-08-26

**Authors:** André DIMAN-RONSONE, Hilary Bienvenue NAMBEI-MBOIFIO, Romaric NZOUMBOU-BOKO, Simon POUNGUINZA, Martin ZÉKANA, Jean de Dieu LONGO, Wilfrid Sylvain NAMBEI

**Affiliations:** 1 Département de sciences biomédicales, Faculté des sciences de la santé, Université de Bangui, République centrafricaine; 2 Laboratoire de Parasitologie, Institut Pasteur de Bangui, République centrafricaine; 3 Laboratoire National de biologie clinique et de santé publique de Bangui, République centrafricaine; 4 Hôpital secondaire de Boali, République centrafricaine; 5 Faculté de Médecine, Pharmacie Odontostomatologie, UCAD, Dakar, Sénégal

**Keywords:** *Plasmodium falciparum*, Accès simple, Traitement, Arthémether-luméfantrine, Artésunate-sulfadoxine-pyriléthamine, Marqueurs de résistance, Diversité génétique, République centrafricaine, Afrique subsaharienne, *Plasmodium falciparum*, Universal access, Treatment, Artemether-lumefantrine, Artesunate-sulfadoxine-pyrimethamine, Resistance markers, Genetic diversity, Central African Republic, Sub-Saharan Africa

## Abstract

**Objectif:**

Surveiller l’efficacité des associations thérapeutiques artémether-luméfantrine (AL) et artésunate-sulfadoxine-pyriméthamine (ASSP) dans le traitement du paludisme simple à *Plasmodium falciparum* chez les enfants de 6 mois à 14 ans à Boali, République centrafricaine.

**Méthode:**

Étude transversale randomisée simple réalisée du 24 août au 27 novembre 2024 chez 199 enfants de 6 mois à 14 ans pris en charge pour accès palustre simple à Boali. Parmi ces enfants, 101 ont été traités par AL et 98 par ASSP avec un suivi pendant 28 jours. La répartition a été faite au hasard. Les réponses ont été classées selon les critères de l’Organisation mondiale de la santé (OMS) en réponses clinique et parasitologique adéquates (RCPA), en échec thérapeutique précoce (ETP), en échec parasitologique tardif (EPT) et en échec clinique tardif (ECT). L’ADN parasitaire a été extrait en utilisant le kit QIAGEN. Les marqueurs de résistance *dhr*, *dhps* et *Kelch 13* ont été analysés par la PCR. Le test de corrélation de Spearman a été utilisé.

**Résultats:**

Pour AL et ASSP, la RCPA était respectivement de 81,8 % et 81,7 %. Il n’y avait pas de différence significative dans la réduction de l’anémie, de la fièvre et la parasitémie entre les deux groupes (p > 0,05). Les mutants *dhfr*-N51I-C59R-S108N-*dhps*-S436A (66,7 %) étaient majoritaires. Le polymorphisme des gènes *K13* a montré 9 mutations synonymes: A621A, A627A, D646D, G496G, G690G, Q707Q, S425S et 4 mutants non synonymes : A578S, V589I, V520I/V637I.

**Conclusion:**

Cette étude montre que des plasmodies porteuses de mutations synonymes et non synonymes ont été détectées, d’où la nécessité de continuer à surveiller régulièrement ces marqueurs de résistance.

## Abstract (en)

**Objective:**

To evaluate the effectiveness of artemether-lumefantrine (AL) and artesunate-sulfadoxine-pyrimethamine (ASSP) for treating uncomplicated *Plasmodium falciparum* malaria in children aged 6 months to 14 years in Boali, Central African Republic.

**Methods:**

From August 24 to November 27, 2024, a simple randomized cross-sectional study was conducted among 199 children aged 6 months to 14 years who were treated for uncomplicated *falciparum* malaria in Boali. One hundred and one children were treated with AL, and 98 with ASSP. All children were followed up for 28 days. Assignment to treatment groups was randomized. Responses were classified according to World Health Organization (WHO) criteria as adequate clinical and parasitological responses (ACPR), early treatment failure (ETF), late parasitological failure (LPF), and late clinical failure (LCF). Parasite DNA was extracted using a QIAGEN kit. The resistance markers *dhfr*, *dhps*, and *kelch13* were analyzed by PCR. Spearman’s correlation test was used.

**Results:**

TThe ADR was 81.8% and 81.7% for AL and ASSP, respectively. There was no significant difference in the reduction of anemia, fever, or parasitemia between the two groups (p > 0.05). The *dhfr*-N51I-C59R-S108N-*dhps*-S436A mutants were the most common (66.7%). Polymorphism in the *K13* genes revealed nine synonymous mutations: A621A, A627A, D646D, G496G, G690G, Q707Q, and S425S. There were also four non-synonymous mutations: A578S, V589I, and V520I/V637I.

**Conclusion:**

This study shows that *Plasmodium* parasites carrying both synonymous and non-synonymous mutations were detected, highlighting the need to regularly monitor these resistance markers.

**Figure:**
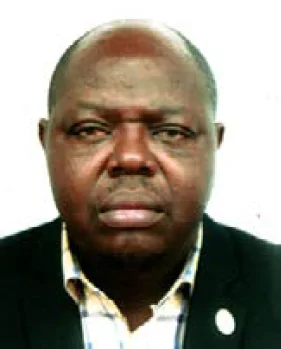

## Introduction

Le paludisme, demeure une des principales causes de morbidité et de mortalité dans le monde. Selon l’Organisation mondiale de la santé (OMS), environ 263 millions de cas, pour un total de 597 000 décès ont été rapportés en 2023 [20]. En République centrafricaine (RCA), la transmission du paludisme est de type pérenne et holoendémique. L’incidence globale du paludisme est de 40,6 cas pour 1000 habitants dans la population générale et de 165,3 cas pour 1000 enfants de moins de 5 ans [7,12]. La lutte contre le paludisme repose, entre autres, sur le diagnostic précoce et le traitement afin de prévenir l’émergence d’une résistance du parasite surtout aux dérivés de l’artémisinine (ACT), traitements de première intention, depuis l’émergence des cas de résistance à la chloroquine (40 %), à l’amodiaquine (23 %) et à la sulfadoxine-pyriméthamine (22 %) [16,17,19]. Les ACT ont été introduits dans la prise en charge du paludisme simple en RCA depuis 2005. Selon les recomman-dations de l’OMS, une surveillance régulière de leur efficacité doit être mise en place à travers les tests d’efficacité thérapeutique dans tous les pays où ces molécules sont utilisées. L’objectif de cette étude était de surveiller l’efficacité des combinaisons arthémether-luméfantrine (AL) et artésunate-sulfadoxine-pyriméthamine (ASSP), utilisées dans le traitement du paludisme simple en RCA, d’une part par la surveillance clinique et parasitologique et d’autre part par l’analyse des marqueurs de résistance au niveau du gène *K13* selon les critères de (i) décroissance parasitaire, (ii) taux des réponses cliniques et parasitologiques adéquates selon les critères de l’OMS [22], (iii) évolution de la température corporelle et amélioration de l’anémie, (iv) polymorphisme génétique du gène *K13* dans les isolats de *Plasmodium falciparum*.

## Méthodologie

Cette étude transversale à visée comparative simple a été réalisée du 24 août au 27 novembre 2024 chez des enfants de 6 mois à 14 ans pris en charge pour accès palustre simple à l’hôpital général de Boali.

Critères d’inclusion: (i) patient âgé de 6 mois à 14 ans souffrant d’une infection mono spécifique à *P. falciparum* avec une parasitémie comprise entre 700 à 200000 formes asexuées/μl de sang; (ii) température axillaire supérieure ou égale à 37,2 °C; (iii) possibilité de suivi avec accès facile au centre de santé; (iv) consentement éclairé des parents. Critères de non-inclusion: (i) présence de signes généraux de danger chez les enfants ou de signes de paludisme à *P. falciparum* grave selon les définitions de l’OMS; (ii) poids corporel inférieur à 5 kg; (iii) taux d’hémoglobine < 8 g/dl; (iv) infestation mixte ou infestation monospécifique par une autre espèce de *Plasmodium*; (v) malnutrition grave; (vi) état fébrile dû à des maladies autres que le paludisme; (vii) antécédents d’hypersensibilité aux traitements administrés.

Critères d’exclusion: (i) patient perdu de vue; (ii) retrait de consentement éclairé; (iii) patient pré-sentant des vomissements persistants; (iv) manifestations d’effets indésirables graves, nécessitant de mettre fin au traitement avant qu’il ne soit achevé; (v) développement d’un paludisme grave dans les 24 heures suivant le début du traitement. Deux associations médicamenteuses ont été utilisées, l’artémether-luméfantrine (AL) et l’artésunate-sulfadoxine-pyriméthamine (ASSP) comme indiqué dans les Tableaux I et II.

**Tableau I:** Posologie de l’artémether-luméfantrine (AL)

Poids	Nombre de comprimés à chacune des 6 prises
5 à < 15 kg	1
15 à < 25 kg	2
25 à < 35 kg	3
≥ 35 kg	4

Nombre de comprimés à chaque prise: 20 mg d'artémether + 120 mg de luméfantrine H0, H8, H24, H36, H48 et H60

**Tableau II:** Posologie de l’artésunate-sulfadoxine-pyriméthamine (ASSP)

Age	Poids	Artésunate (J1-J3)	SP (dose unique J1)
< 1 an	5-8 kg	½ cp (25 mg) x1/jour	¼ cp
1-5 ans	9-17 kg	1 cp (50 mg) x1/jour	½ cp
6-9 ans	18-29 kg	2 cp (100 mg) x1/jour	1 cp
10-14 ans	30-39 kg	3 cp (150 mg) x1/jour	1,5 cp
≥ 15 ans-adultes	≥ 40 kg	4 cp (200 mg) x1/jour	2 cp

Artésunate 50 mg + 500 mg de sulfadoxine + 25 mg de pyriméthamineArtesunate 50 mg + 500 mg sulfadoxine + 25 mg pyrimethamine

La taille de chaque bras de l’étude (N) a été calculée en utilisant la formule N=Z^2^*P (1-P) / E^2^. La proportion d’échec anticipé dans la population (P) était de 15 %, le niveau de confiance (Z) de 95 %, la marge d’erreur (E) de 10 % par bras [21]. Le nombre minimum de patients nécessaire était alors de 88 par traitement.

Un infirmier procédait à la prise du poids et de la température axillaire. La recherche des signes de gravité était réalisée par le clinicien. Un technicien effectuait une goutte épaisse (GE), un frottis mince et un dosage de l’hémoglobine (Hemocue HB 301 analyser, Kuvettgatan, Suède). Les enfants ayant un taux d’hémoglobine supérieur à 8 g/dl et une GE comprise entre 700 et 200 000 parasites/μl étaient inclus après accord du responsable.

Un numéro de code a été attribué à chaque patient à J0 de façon aléatoire en vue de rendre les dossiers anonymes. Un coefficient entre 0 et 1 a été généré sur fichier Excel 2013, pour répartir aléatoirement les patients dans 2 différents groupes de traitements. La première dose du traitement a été administrée au Centre de santé par le personnel soignant, et les patients ont été observés pendant 30 minutes avant de quitter le site. En cas de vomissements, la même dose était répétée. Les doses suivantes étaient administrées à domicile par les responsables en charge de l’enfant. L’adhésion au traitement était vérifiée par le personnel soignant le 3e jour sur présentation du reste de la plaquette et description de l’administration du traitement. Afin d’apprécier les clairances parasitaire et thermique, une GE et une prise de la température étaient réalisées à J3, J7, J14, J21 et J28.

Les critères d’évaluation des réponses étaient ceux définis par l’OMS [21]. Les réponses au traitement ont été classées en réponse clinique parasitologique adéquate (RCPA), échec thérapeutique précoce (ETP), en échec clinique tardif (ECT) et échec parasitologique tardif (EPT) comme suit : (i) ETP, de J0 à J3, signe de danger ou d’accès palustre à J1, J2 ou J3; fièvre + parasitémie augmentée à J2 ou fièvre + parasitémie à J3; ou parasitémie à J3 supérieure ou égale à 25% de la parasitémie à J0, (ii) EPT, de J7 à J14 ou J28, présence des parasites sans fièvre, (iii) ECT, de J4 à J14 ou J28, signes de dangers ou d’accès palustre après J3; fièvre + parasitémie positive entre J4 et J28, (iv) RCPA, absence de parasites détectables entre J2 et J28, et absence de fièvre entre J2 et J28.

Les observations microscopiques étaient faites par deux microscopistes en double lecture. Les densités parasitaires étaient exprimées selon la formule : nombre de parasites asexués x 8000/ nombre de globules blancs par µl de sang sur 200 champs de leucocytes. Une lame sur 3 a fait l’objet d’une 2e lecture par un troisième microscopiste accrédité par l’Institut Pasteur de Bangui pour le contrôle qualité, sans qu’aucune discordance n’ait été constatée.

Pour les analyses moléculaires, cinq spots de sang ont été réalisés sur papier filtre Whatman. Parmi les 199 échantillons collectés pour la réalisation de la PCR espèce pour valider la présence de *P. falciparum* puis séquençage des gènes *K13*, *dhps* et *dhfr,* 73 étaient négatifs soit 36,7% et 126 étaient positifs soit 63,3%. Pour le séquençage, les 126 échantillons ont donné des résultats exploitables pour le gène *K13*, 123 pour *dhfr* et 114 sont exploitables pour *dhps*.

L’ADN parasitaire a été extrait par QIAGEN (QIAamp DNA Blood Minikit) [1]. La PCR en temps réel à l’aide de SYBR et d’amorces spécifiques pour le gène cytochrome B de *P. falciparum* a été réalisée pour confirmer l’espèce plasmodiale infectante à J0 [22]. Les réactifs commercialisés dans le kit SYBR Green Master Mix I (Roche Diagnostics) comprennent deux mélanges 1a et 1b contenant l’intercalant fluorescent SYBR green, les dNTP, la Taq polymérase et le mgCl_2_. Les réactions ont lieu dans le thermo-cycleur Lightcycler et sous un volume final de 20 µl dont 5 µl d’ADN extrait à amplifier. Un couple d’amorces spécifiques de *P. falciparum :* PanPalu 5’-AGTGTGTATCAATCGAGTTTC-3’ et PfaIR 5’-AGTTCCCCTAGAATAGTTACA-3’ était utilisé. Les échantillons positifs ont été sélectionnés pour l’analyse des marqueurs de résistance à l’artémisinine (*K13*), à la pyriméthamine (*dhfr*) et à la sulfadoxine (*dhps*) avec les amorces spécifiques présentées dans le tableau III [23-25].

**Tableau III:** Séquences des amorces spécifiques pour les gènes de résistance étudiés

	Gène K13 (5'-3')	Gène dhfr (5'-3')	Gène dhps (5'-3')
F	GCCAAGCTGCCATTCATTTG	TTTATATTTTCTCCTTTTTA	CCATTCCTCATGTGTATACAACAC
R	GCCTTGTTGAAAGAAGCAGA	CATTTTATTATTCGTTTTCT	CTTGGTCTATTTTTGTTAAAACATCC

F : amorces sens; R : amorces anti sens

Les régions qui codaient pour ces gènes étaient amplifiées et séquencées par *amplicon sequencing* à l’Institut Pasteur de Madagascar [23-25].

Le programme d’amplification par PCR avec le *sybr green* en temps réel est consigné dans le tableau IV.

**Tableau IV:** Programme de PCR en temps réel pour l’amplification de gène cytochrome B de *Plasmodium falciparum*

	Étapes	Temps et températures
	Dénaturation initiale	8 minutes à 95°C
Cycles d'amplifications répétés 40 fois / Amplification cycles repeated 40 times	Dénaturation	10 secondes à 95°C
	Hybridation des amorces	10 secondes à 58°C
	Élongation	30 secondes à 72°C
Courbes de fusions / Melting curves	Dénaturation	2 secondes à 95°C
	Hybridation	20 secondes à 50°C
	Dénaturation progressive	0,2°C/seconde jusqu'à 95°C
	Refroidissement	30 secondes à 95°C

Les moyennes sont indiquées avec des intervalles de confiance à 95% et les valeurs extrêmes de la distribution.

Les tests statistiques utilisés pour comparer moyennes et proportions utilisaient le seuil de p< 0,05. Afin d’établir un lien entre les marqueurs de résistance et les paramètres cliniques et biologiques, le coefficient de corrélation de Pearson a été utilisé avec rs > 0 considéré comme corrélation positive.

## Résultats

Au total, 199 patients ont été inclus dont 101 pour AL et 98 pour ASSP. La moyenne d’âge était de 3,76 ± 1,58 ans avec des extrêmes de 6 mois à 14 ans pour AL et de 4,37 ± 0,58 ans avec des extrêmes de 6 mois à 13 ans pour ASSP sans différence significative entre les deux groupes (p = 0,73). Le sex-ratio était de 1,29. La température moyenne initiale était de 37,74 ± 1,02 °C pour AL avec des extrêmes de 37,9 °C à 39,6 °C et de 37,75 ± 0,9 °C pour ASSP avec des extrêmes de 37,6 °C à 39,6 °C. La parasitémie moyenne était de 4 216,39 ± 6 700 parasites/μl pour AL avec des extrêmes de 740 à 58960 parasites/ml et 2567,98 parasites/ml pour ASSP avec des extrêmes de 720 à 68 940 sans différence significative entre les deux groupes (p = 0,64). Le taux moyen de l’hémoglobine était de 11,58 ± 0,78 g/dl pour AL et 11,59 ± 0,88 g/dl pour ASSP (p = 0,9) (Tableau 5). A J3, 97 patients (96 %) avaient une parasitémie négative pour AL et 94 patients (95,9 %) avaient une parasitémie négative pour ASSP. La situation était restée stable à J7, J14, J21 et J28 (Fig. 1).

**Figure 1:**
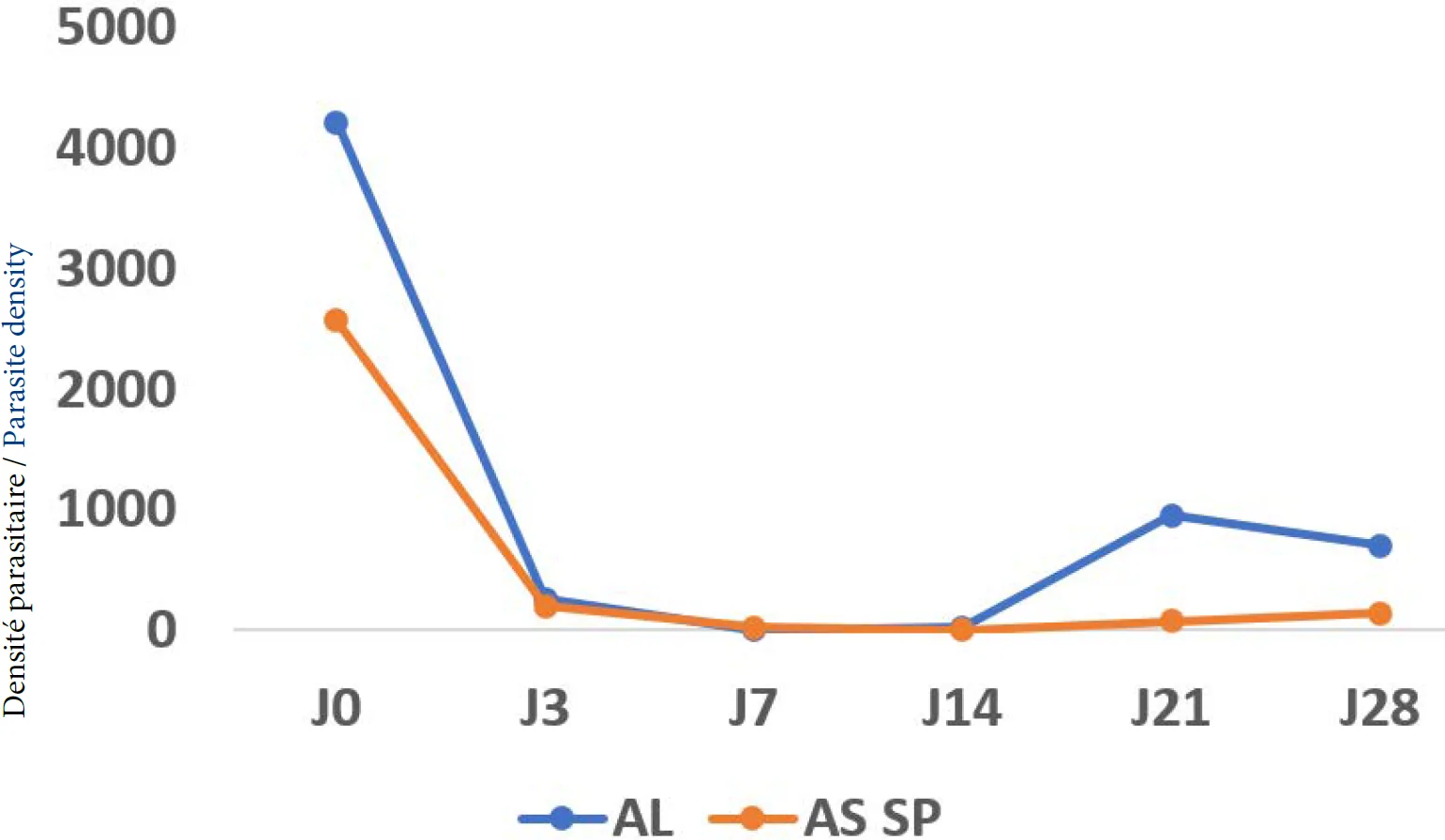
Décroissance parasitaire au cours du suivi selon le traitement de J0 à J28

**Tableau V:** Caractéristiques sociodémographiques et biologiques de la population d’étude à l’inclusion

Caractéristiques		AL N = 101 (%)	ASSP N = 98 (%)	Total N = 199	p
Sexe	Masculin	54 (53,5)	58 (59,6)	112 (56,3)	0,25
	Féminin	47 (46,5)	40 (40,8)	87 (43,7)	
Âge (an)	Moyenne	3,76±1,58	4,37±0,58	199	0,73
	Extrême	6 mois - 14 ans	6 mois - 13 ans		
Parasitémie /μl de sang	Moyenne	4 216,39 ± 6 700	2 567,98 ± 4 300	3 404, 61	0,53
	Extrême	740 – 58 960	720 – 68 940		
Hémoglobine (g/dl)	Moyenne	11,58±0,78	11,59±0,88	11,58	0,90
Température	Moyenne	37,74 ± 1,02	37,75 ± 0,9	37,74	0,64
	Extrême	37,90-39,60	37,60-39,60		

AL: artémether-luméfantrine, ASSP : artésunate-sulfaméthoxypyrazine-pyriméthamine

A J3, 100 % des patients avaient une température normale pour AL et ASSP et la situation était restée stable à J7, J14, J21 et J28.

à J3, le taux de guérison était de 98,8 % pour AL et ASSP. A J28, le nombre total de patients *per protocole* était encore de 159. Les perdus de vue étaient au nombre de 19 pour AL et 21 pour ASSP sans différence significative entre les 2 groupes (p> 0,05). Aucun patient n’a été exclu de l’étude pour cause de manifestations d’effets indésirables graves, nécessitant de mettre fin au traitement avant qu’il ne soit achevé. Nous avons observé avec AL : un cas d’ETP (1,2 %), trois cas d’ECT (3,7 %) et 11 cas d’EPT (13,4 %). Avec ASSP, nous avons relevé deux cas d’ETP (2,7 %) et 12 cas d’EPT (15,6 %). Par ailleurs, nous avons observé 130 cas de RCPA cumulés (81,8 %) [IC95%: 63,8-72,2] dont 67 cas pour AL (81,8 %) et 63 cas pour ASSP (81,9 %) sans différence statistiquement significative entre les deux groupes (p> 0,05) (Tableau VI).

**Tableau VI:** Réponses cliniques et biologiques aux traitements par artémether-luméfantrine (AL) et artésunate / sulfadoxine-pyriméthamine (ASSP)

Paramètres de suivi J3 à J28	AL N = 82 (%)	ASSP N = 77 (%)	p
Taux de guérison à J3	82 (100)	75 (97,40)	0,19
ETP	1 (1,22)	2 (2,60)	0,39
ECT	3 (3,67)	0 (0,00)	0,23
EPT	11 (13,41)	12 (15,58)	0,85
RCPA	67 (81,70)	63 (81,82)	0,20
**Total per protocole**	**82 (100)**	**77 (100)**	

Parmi les 199 échantillons analysés, 63,31% soit 126/199 échantillons ont donné des résultats exploitables pour le séquençage des gènes *K13*, 61,8% soit 123/199 pour le gène *dhfr* et 57,28% soit 114/199 pour le gène *dhps*.

Pour le gène *K13*, l’allèle sauvage a été détecté dans 111 échantillons soit 87,40%. Neuf mutations synonymes ont été trouvées à savoir : A621A, A627A, D646D, G496G, G690G, Q707Q, S425S, T535T, V510V avec une prévalence variant entre 0,8 et 1,6 %. Quatre mutations non synonymes ont été détectées à savoir : A578S (1,6 %), V589I (0,8 %) ainsi que V520I associé à V637I dans deux échantillons (1,6 %). Aucune mutation considérée comme associée à la baisse de l’efficacité de l’arté-misinine n’a été observée au cours de cette étude (Fig. 2). Le mutant non synonyme V589I sur le gène *K13* a été associé à un retard de clairance parasitaire (rs = 0,18).

**Figure 2:**
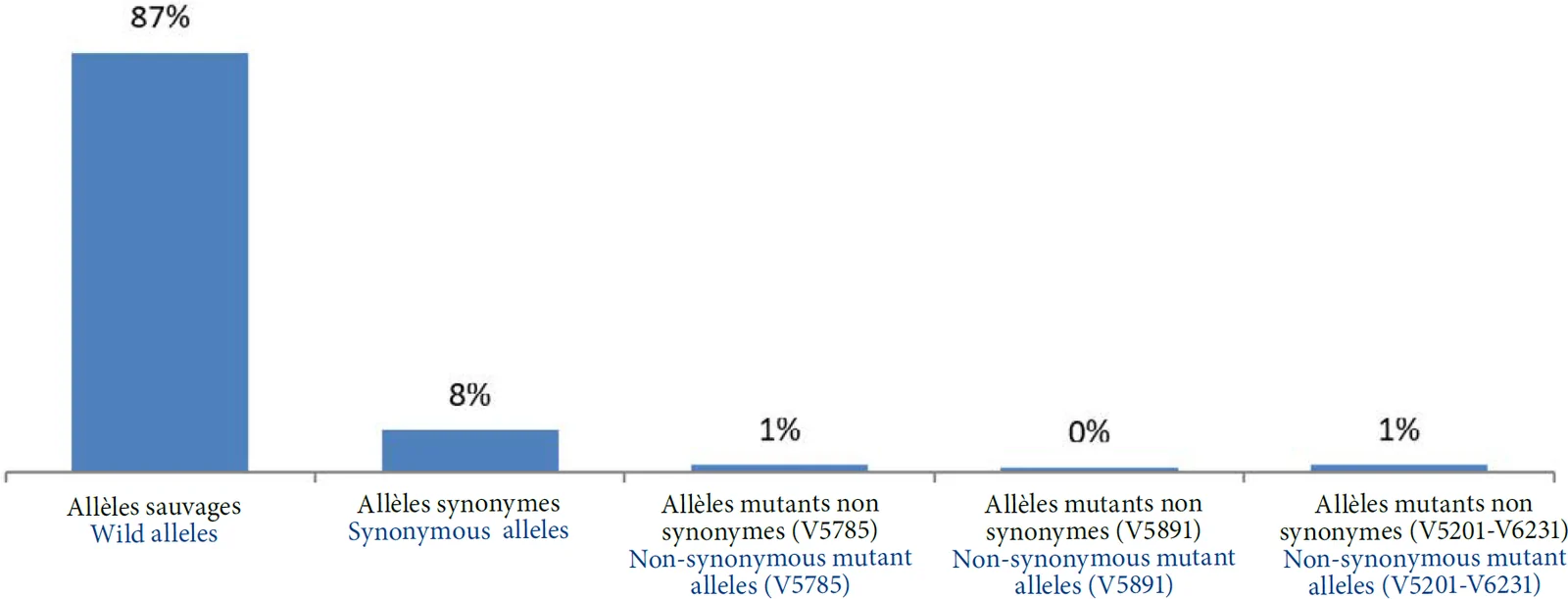
Proportions des allèles sauvages et mutants du gène *Kelch13 propeller*

L’analyse du gène *dhfr* a montré que tous les échantillons analysés comportaient des mutations. Le triple mutant N51I-C59R-S108N a été détecté dans 95,1 % des cas, le double mutant C59R-S108N dans 3,2 % et le double mutant N51I-S108N dans 1,7 % des cas (Fig. 3).

**Figure 3:**
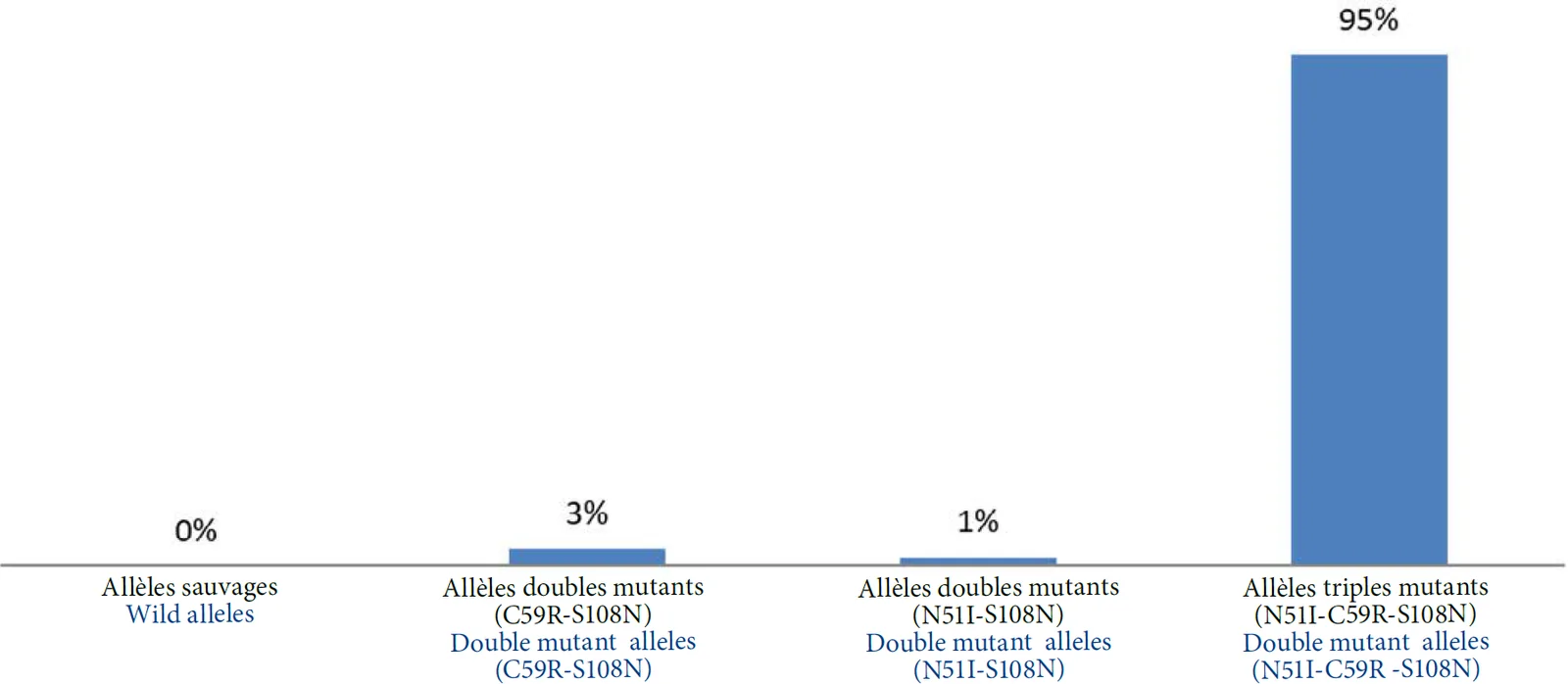
Proportions des allèles sauvages et mutants du gène *dhfr* résistant à la Pyriméthamine

Le gène *dhps* présentait des allèles sauvages dans 28 isolats, soit 24,6 % des cas. Le mutant S436A a été retrouvé dans 79 isolats soit 69,3 % des cas et les allèles mutants I431V-S436A, S436A-A581G et S436A-A613S dans 1,8 %, 0,9 % et 0,9 % des cas respectivement. Les mutants I431V-S436A-A581G_ A613S ont été retrouvés dans 2,7 % des cas (Fig. 4). Par ailleurs, des mutations sur les gènes *dhfr* et *dhps* au sein d’un même échantillon ont été détectées 86 fois (75,4 %). Le mutant *dhfr*-N51I-C59R-S108N-*dhps*-S436A était majoritaire (66,6 % des cas). La mutation sur le codon 540 qui détermine la résistance à la sulfadoxine n’a pas été retrouvée au cours de cette étude.

**Figure 4:**
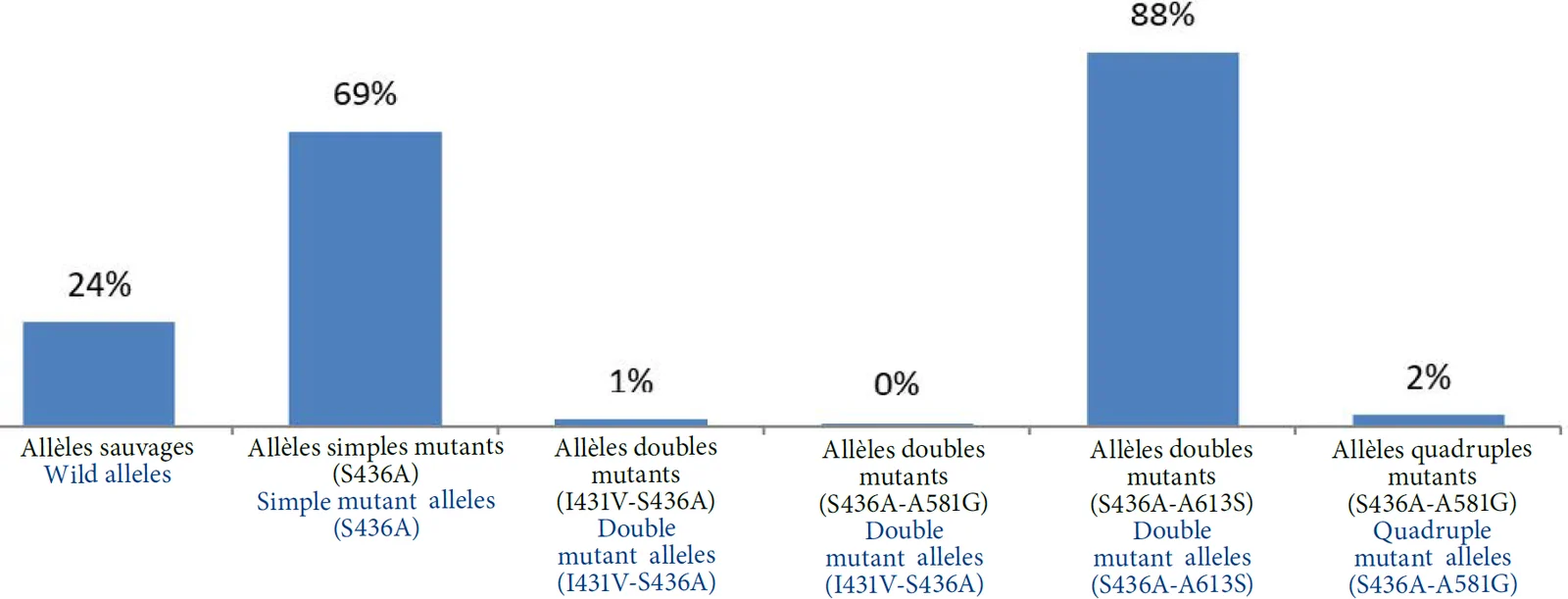
Proportions des allèles sauvages et mutants du gène *dhps* résistant à la sulfadoxine

## Discussion

La surveillance régulière des combinaisons thérapeutiques à base d’artémisinine constitue un outil nécessaire pour prévenir l’émergence et la propagation de la résistance aux antipaludéens. Ainsi, notre travail à Boali montre que la réduction attendue, rapide et nette de la parasitémie après trois jours de traitement par AL et ASSP à plus de 95 % dans les deux groupes de traitement sans différence significative entre les deux groupes (p > 0,05) était encore obtenue. Il en était de même pour la fièvre et l’anémie. Par ailleurs, l’analyse du polymorphisme génétique au niveau du gène *K13* a montré que la majorité des isolats contenait des allèles de type sauvage (87,4 %) et qu’aucune mutation considérée comme associée à la résistance aux dérivés de l’artémisinine n’a été observée. Ces résultats confortent l’utilisation de ces deux combinaisons comme traitement de première intention dans cette région du pays. Toutefois, le suivi long des patients dans notre contexte constitue une limite dans la mesure où on note un nombre élevé de perdu de vue (PDV) dans les deux groupes. Ce qui réduit la taille de l’échantillon nécessaire pour une analyse approfondie et constitue un biais majeur dans la représentativité et la sélection des patients. Néanmoins, le calcul de la taille de l’échantillon prenant en compte par anticipation un risque de 10% de PDV est un atout pour la validité de ce travail. De même, le suivi à J28 peut sousestimer les échecs tardifs, en particulier pour les molécules à demivie prolongée. Nos résultats corroborent ceux rapportés antérieurement et réalisés dans différents pays endémiques [2-6,8-11,13-19]. Ces résultats plaident en faveur de la poursuite de l’utilisation des combinaisons à base des dérivés de l’artémisinine.

De toutes les mutations non synonymes, seule la mutation V589I était considérée comme associée au fait que certains patients éliminent moins vite les parasites sous traitement AL et ASSP (rs = 0,18). Ce qui diffère de certains travaux en Afrique [2,4]. Cette différence pourrait s’expliquer par des approches méthodologiques différentes. La mutation non synonyme A578S rapportée au cours de ces travaux a également été observée au Mali avec la même proportion de 1,5 % [2]. Ces mutations ne font pas partie des marqueurs de résistance validés ou candidats associés à la résistance signalés par l’OMS [8,21]. Ces mutations traduisent la diversité génétique du gène *K13* et ont été rapportées au Niger [5], au Kenya [6], en Guinée, en RDC [15] et au Sénégal [10]. Cependant, les cas d’échecs observés avec ASSP sont dus à des mutations observées au niveau des codons 51, 59 et 108 du gènes *dhfr*, et des codons 431, 436 et 581 du gène *dhps*. Ces mêmes résultats ont été rapportés dans des travaux antérieurs [14]. L’absence de mutation sur le codon 540 plaide en faveur de l’efficacité de la sulfadoxine et donc de l’association ASSP.

## Conclusion

L’étude confirme l’efficacité clinique et biologique des ACT chez les enfants à Boali en 2024. L’analyse du gène *K13* a majoritairement mis en évidence les allèles de type sauvage. Les mutations synonymes et non synonymes observées traduisent la diversité génétique du gène *K13* et ne sont pas associées à la résistance aux dérivés de l’artémisinine. Il serait judicieux de continuer à surveiller régulièrement l’efficacité des dérivés de l’artémisinine en vue de réduire la transmission de la maladie et prévenir l’émergence de la résistance.

## Remerciements

Nous remercions les participants pour leur coo-pération au cours de cette étude ainsi que le personnel de l’hôpital secondaire de Boali pour leurs appuis à la réussite de nos travaux.

## Financement

Ce travail a été réalisé grâce à l’appui financier et technique du Fonds Mondial et de l’Organisation mondiale de la santé (OMS).

## Contribution des auteurs et autrices

DRA : collecte des données et analyse de laboratoire

NMHB : lecture et correction du manuscrit NBR : analyse des données

SP : collecte des données MZ : collecte des données

JDL : lecture et corrections du manuscrit

WSN: conception, élaboration, analyse des don-nées, validation des résultats, rédaction et sou-mission du manuscrit

## Déclaration de liens d’intérêt

Aucun lien d’intérêt n’a été déclaré.
