# Supplementary material for: Monitoring the efficacy of artemether-lumefantrine and artesunate-sulfadoxine/pyrimethamine combinations for treating uncomplicated Plasmodium falciparum malaria in Boali, Central African Republic
Source: Med Trop Sante Int. 2026 Aug 26;6(3):mtsi.v6i3.2026.883. [Article in French] doi: 10.48327/mtsi.v6i3.2026.883 (PMC13697912; doi:10.48327/mtsi.v6i3.2026.883)
Supplement: Related texts

# Research article: Monitoring the efficacy of artemether-lumefantrine and artesunate-sulfadoxine/pyrimethamine combinations for treating uncomplicated *Plasmodium falciparum* malaria in Boali, Central African Republic

## Introduction

Malaria remains one of the leading causes of morbidity and mortality worldwide. According to the World Health Organization (WHO), there were approximately 263 million reported cases in 2023, resulting in 597,000 deaths [20]. In the Central African Republic (CAR), malaria transmission is year-round and holoendemic. The overall incidence is 40.6 cases per 1,000 people in the general population and 165.3 cases per 1,000 children under five years of age [7,12]. The fight against malaria relies on early diagnosis and treatment to prevent the emergence of parasite resistance, particularly to artemisinin-based combination therapies (ACTs), the first-line treatment, since resistance has emerged to chloroquine (40%), amodiaquine (23%), and sulfadoxine-pyrimethamine (22%) [16,17,19]. ACTs have been used to manage uncomplicated malaria in CAR since 2005. According to WHO recommendations, all countries where these drugs are used must implement regular monitoring of their effectiveness through therapeutic effectiveness testing. This study aimed to monitor the effectiveness of artemether-lumefantrine (AL) and artesunate-sulfadoxine-pyrimethamine (ASSP) combinations used to treat uncomplicated malaria in the CAR. Monitoring was conducted through clinical and parasitological surveillance and analysis of resistance markers at the *K13* gene level based on the following criteria: (i) parasite decline; (ii) rates of adequate clinical and parasitological responses according to WHO criteria [22]; (iii) changes in body temperature and improvement in anemia; and (iv) genetic polymorphism of the *K13* gene in *Plasmodium falciparum* isolates.

## Methodology

This simple, cross-sectional, comparative study was conducted from August 24 to November 27, 2024. It was conducted among children aged six months to 14 years who were treated for uncomplicated malaria at Boali General Hospital.

Inclusion criteria: (i) Patients aged 6 months to 14 years with monospecific *P. falciparum* infection and parasitemia ranging from 700 to 200,000 asexual forms per μL of blood, (ii) an axillary temperature of at least 37.2°C, (iii) the ability to undergo follow-up with easy access to the health center, and (iv) informed consent from parents.

Non-inclusion criteria: (i) the presence of general danger signs in children or signs of severe *P. falciparum* malaria, according to WHO definitions; (ii) body weight less than 5 kg; (iii) hemoglobin level less than 8 g/dL; (iv) mixed infection or monospecific infection with another Plasmodium species; (v) severe malnutrition; (vi) a febrile illness due to a condition other than malaria; and (vii) a history of hypersensitivity to the administered treatments.

Exclusion criteria: (i) patient lost to follow-up; (ii) withdrawal of informed consent; (iii) patient with persistent vomiting; (iv) manifestations of serious adverse effects requiring discontinuation of treatment before completion; and (v) development of severe malaria within 24 hours of treatment initiation.

Two drug combinations were used: artemether-lumefantrine (AL) and artesunate-sulfadoxine-pyrimethamine (ASSP), as shown in Tables I and II.

**Table I:** Dosage of artemether-lumefantrine (AL)

Weight	Number of tablets for each of the 6 doses
5 to < 15 kg	1
15 to < 25 kg	2
25 to < 35 kg	3
≥ 35 kg	4

Number of tablets per dose: 20 mg of artemether + 120 mg of lumefantrine H0, H8, H24, H36, H48, and H60

**Table II:** Dosage of artesunate-sulfadoxine-pyrimethamine (ASSP)

Age	Weight	Artesunate (D1-D3)	SP (single dose D1)
< 1 year	5-8 kg	½ tab (25 mg) x1/day	¼ tab
1-5 years	9-17 kg	1 tab (50) x1/day	½ tab
6-9 years	18-29 kg	2 tab (100) x1/day	1 tab
10-14 years	30-39 kg	3 tab (150) x1/day	1.5 tab
≥ 15 years-adults	≥ 40 kg	4 tab (200) x1/day	2 tab

Artesunate 50 mg + 500 mg sulfadoxine + 25 mg pyrimethamine

The sample size for each study arm (N) was calculated using the formula N = Z²*P(1-P)/E². The expected failure rate in the population (P) was 15%, the confidence level (Z) was 95%, and the margin of error (E) was 10% per arm [21]. Therefore, the minimum number of patients required was 88 per treatment.

A nurse measured weight and axillary temperature. The clinician assessed signs of severity. A technician performed a thick smear, a thin smear, and a hemoglobin measurement using a Hemocue HB 301 analyzer (Kuvettgatan, Sweden). After approval by the principal investigator, children with a hemoglobin level greater than 8 g/dL and a thick smear count between 700 and 200,000 parasites/μL were included.

On D0, a code number was randomly assigned to each patient to anonymize the records. In Excel 2013, a coefficient between 0 and 1 was generated to randomly assign patients to two different treatment groups. Healthcare staff administered the first dose of treatment at the health center, and patients were observed for 30 minutes before leaving the site. If a patient vomited, the same dose was repeated. Subsequent doses were administered at home by the caregivers responsible for the child. Healthcare staff verified adherence to treatment on the third day by having patients present the remaining blister pack and describe how the treatment was administered.

To assess parasite clearance and fever resolution, stool examinations and temperature measurements were performed on D3, D7, D14, D21, and D28.

The WHO [21] defined the criteria for evaluating treatment responses. Treatment responses were classified in adequate clinical and parasitological response (ACPR), early treatment failure (ETF), late clinical failure (LCF), late parasitological failure (LPF), as follows: (i) ETF, from D0 to D3: signs of danger or malaria symptoms on D1, D2, or D3; fever plus increased parasitemia on D2; fever plus parasitemia on D3; or parasitemia on D3 equal to or greater than 25% of D0 parasitemia, (ii) LPF, from D0 to D7, 14, or D28: presence of parasites without fever, (iii) LCF, from D0 to D4, D14, or D28: signs of danger or malaria symptoms after D3; fever plus positive parasitemia between D4 and D28, and (iv) ACPR: absence of detectable parasites between D2 and D28 and absence of fever between D2 and D28.

Two microscopists performed dual microscopic readings. Parasite densities were calculated using the following formula: number of asexual parasites × 8,000 / number of white blood cells per µL of blood across 200 leukocyte fields. One out of every three slides was reviewed a second time by a third microscopist, accredited by the Pasteur Institute of Bangui, for quality control; no discrepancies were observed.

For molecular analyses, five blood spots were prepared on Whatman filter paper. Of the 199 samples collected for species-specific PCR to confirm the presence of *P. falciparum*, followed by sequencing of the *K13*, *dhps*, and *dhfr* genes, 73 were negative (36.7%) and 126 were positive (63.3%). The 126 positive samples yielded usable sequencing results for the *K13* gene in 123 samples, the *dhfr* gene in 114 samples, and the *dhps* gene in 114 samples.

Parasite DNA was extracted using the QIAGEN QIAamp DNA Blood Minikit [1]. Realtime PCR using SYBR Green and primers specific for the cytochrome B gene of P*. falciparum* was performed to confirm the infecting *Plasmodium* species at D0 [22]. The reagents supplied in the SYBR Green Master Mix I kit (Roche Diagnostics) include two mixtures, 1a and 1b, containing the fluorescent intercalator SYBR Green, dNTPs, Taq polymerase, andmgCl_2_. Reactions were performed in a LightCycler thermocycler at a final volume of 20 µl, including 5 µl of extracted DNA to be amplified. A pair of primers specific to *P. falciparum*—PanPalu 5’-AGTGTGTATCAATCGAGTTTC-3’ and PfaIR 5’-AGTTCCCCTAGAATAGTTACA-3’—was used. Positive samples were selected for analysis of resistance markers for artemisinin (*K13*), pyrimethamine (*dhfr*), and sulfadoxine (*dhps*) using the specific primers presented in Table III [23–25]. The regions encoding these genes were amplified and sequenced by amplicon sequencing at the Pasteur Institute of Madagascar [23–25].

**Table III:** Sequences of specific primers for the resistance genes studied

	K13 gene (5'-3')	dhfr gene (5'-3')	dhps gene (5'-3')
F	GCCAAGCTGCCATTCATTTG	TTTATATTTTCTCCTTTTTA	CCATTCCTCATGTGTATACAACAC
R	GCCTTGTTGAAAGAAGCAGA	CATTTTATTATTCGTTTTCT	CTTGGTCTATTTTTGTTAAAACATCC

F : sense primers; R : antisense primers

The PCR amplification protocol using real-time SYBR Green is presented in Table IV.

**Table IV:** Real-time PCR program for amplification of the cytochrome B gene of Plasmodium falciparum

	Steps	Time and temperature
	Initial denaturation	8 minutes at 95°C
Amplification cycles repeated 40 times	Denaturation	10 seconds at 95°C
	Primer hybridization	10 seconds at 58°C
	Melting	30 seconds at 95°C
Melting curves	Denaturation	2 seconds at 95°C
	Extension	20 seconds at 50°C
	Gradual denaturation	0.2 second at 95°C
	Cooling	30 seconds at 95°C

Means are reported with 95% confidence intervals and the extreme values of the distribution.

Statistical tests used to compare means and pro-portions employed a p-value threshold of p < 0.05. To establish a relationship between resistance markers and clinical and biological parameters, Pearson’s correlation coefficient was used, with rs > 0 considered a positive correlation.

## Results

A total of 199 patients were included in the study, comprising 101 with AL and 98 with ASSP. The mean age was 3.76 ± 1.58 years for AL and 4.37 ± 0.58 years for ASSP. Ages ranged from six months to 14 years for AL and six months to 13 years for ASSP. There was no significant difference between the two groups (p = 0.73). The sex ratio was 1.29. The mean initial temperature was 37.74 ± 1.02°C for AL, ranging from 37.9°C to 39.6°C, and 37.75 ± 0.9°C for ASSP, ranging from 37.6°C to 39.6°C. The mean parasitemia was 4,216.39 ± 6,700 parasites/μL for AL, ranging from 740 to 58,960 parasites/μL, and 2,567.98 parasites/ μL for ASSP, ranging from 720 to 68,940. There was no significant difference between the two groups (p = 0.64). The mean hemoglobin level was 11.58 ± 0.78 g/dL for AL and 11.59 ± 0.88 g/ dL for ASSP (p = 0.9) (Table V).

**Table V:** Sociodemographic and biological characteristics of the study population at inclusion

Characteristics		AL N = 101 (%)	ASSP N = 98 (%)	Total N = 199	p
Gender	Male	54 (53,5)	58 (59,6)	112 (56,3)	0,25
	Female	47 (46,5)	40 (40,8)	87 (43,7)	
Age (years)	Average	3,76±1,58	4,37±0,58	199	0,73
	Extreme	6 months - 14 years	6 months - 13 years		
Parasitemia /μl of blood	Average	4 216,39 ± 6 700	2 567,98 ± 4 300	3 404, 61	0,53
	Extreme	740 – 58 960	720 – 68 940		
Hemoglobin (g/dl)	Average	11,58±0,78	11,59±0,88	11,58	0,90
Temperature	Average	37,74 ± 1,02	37,75 ± 0,9	37,74	0,64
	Extreme	37,90-39,60	37,60-39,60		

AL: artemether-lumefantrine, ASSP: artesunate-sulfadoxine-pyrimethamine

On D3, 97 patients (96%) in the AL group and 94 patients (95.9%) in the ASSP group had negative parasitemia. This remained stable on D7, D14, D21, and D28 (Fig. 1).

**Figure 1:** Parasitic decacy to treatments from D0 to D28

One hundred percent of patients had normal temperatures on D3 for both AL and ASSP, and this remained stable on D7, D14, D21, and D28.

The cure rate was 98.8% for both AL and ASSP on D3. On D28, the total number of patients per protocol remained at 159. There were 19 patients lost to follow-up in the AL group and 21 in the ASSP group, with no significant difference be-tween the two groups (p > 0.05). No patients were excluded from the study due to serious adverse events requiring discontinuation of treatment before completion. With AL, we observed one case of ETF (1.2%), three cases of LCF (3.7%), and 11 cases of LPF (13.4%). With ASSP, we recorded two cases of ETF (2.7%) and 12 cases of LPF (15.6%). Additionally, we observed a cumulative total of 130 RCPA cases (81.8%) [95% CI: 63.8–72.2], including 67 AL cases (81.8%) and 63 ASSP cases (81.9%), with no significant difference between the groups (p > 0.05) (Table VI).

**Table VI:** Clinical and biological responses to artemether-lumefantrine (AL) and artesunate/sulfadoxine-pyrimethamine (ASSP)

AL N = 82 (%)	AL N = 82 (%)	ASSP N = 77 (%)	p
Recovery rate on D3	82 (100)	75 (97,40)	0,19
ETF	1 (1,22)	2 (2,60)	0,39
LCF	3 (3,67)	0 (0,00)	0,23
LPF	11 (13,41)	12 (15,58)	0,85
ACPR	67 (81,70)	63 (81,82)	0,20
**Total per protocol**	**82 (100)**	**77 (100)**	

Of the 199 samples analyzed, 63.31% (126 samples) yielded usable results for *K13* gene sequencing, 61.8% (123 samples) for the *dhfr* gene, and 57.28% (114 samples) for the *dhps* gene.

The wild-type allele was detected in 111 sam-ples (87.40%) for the *K13* gene. Nine synony-mous mutations were identified: A621A, A627A, D646D, G496G, G690G, Q707Q, S425S, T535T, and V510V. These mutations had a prevalence ranging from 0.8% to 1.6%. Four non-synony-mous mutations were detected: A578S (1.6%), V589I (0.8%), and V520I, which was associated with V637I in two samples (1.6%). No mutations associated with reduced artemisinin efficacy were observed during this study (Fig. 2). The non-synonymous mutation V589I in the *K13* gene was associated with delayed parasite clearance (rs = 0.18).

**Figure 2:** Proportions of wild and mutant alleles of the *Kelch13 propeller gene*

Analysis of the *dhfr* gene showed that all analyzed samples contained mutations. The triple mutant N51I-C59R-S108N was present in 95.1% of cases; the double mutant C59R-S108N was present in 3.2% of cases; and the double mutant N51I-S108N was present in 1.7% of cases (Fig. 3).

**Figure 3:** Proportions of wild and mutant alleles of the *dhfr* gene resistant to pyrimethamine

Wild-type alleles were exhibited by the *dhps* gene in 28 isolates, representing 24.6% of cases. The S436A mutant was found in 79 isolates (69.3% of cases), and the I431V-S436A, S436A-A581G, and S436A-A613S mutant alleles were found in 1.8%, 0.9%, and 0.9% of cases, respectively (Fig. 4). The I431V-S436A-A581G-A613S mutant was found in 2.7% of cases (Fig. 5). Additionally, mutations in both the *dhfr* and *dhps* genes within the same sample were detected 86 times (75.4%). The *dhfr*-N51I-C59R-S108N-*dhps*-S436A mutant was the most common, accounting for 66.6% of cases. The mutation at codon 540, which confers resistance to sulfadoxine, was not detected during this study.

**Figure 4:** Proportion of wild and mutant alleles of the *dhps* gene resistant to sulfadoxin

## Discussion

Regularly monitoring artemisinin-based com-bination therapies is necessary to prevent the emergence and spread of resistance to antimalarial drugs. Our study in Boali shows that the expected rapid and significant reduction in parasitemia by more than 95% after three days of treatment with AL and ASSP was achieved in both treatment groups, with no significant difference between the two groups (p > 0.05). The same was true for fever and anemia. Furthermore, analysis of genetic polymorphism in the *K13* gene revealed that most isolates contained wild-type alleles (87.40%), and no mutations associated with artemisinin resistance were observed. These results support the use of these two combinations as first-line treatments in this region. However, a limitation of our study is the high number of patients lost to follow-up (LTFU) in both groups. This reduces the sample size required for an in-depth analysis and constitutes a major source of bias in the representativeness and selection of patients. Nevertheless, the sample size calculation, which anticipated a 10% risk of LTFU, strengthens the validity of this study. Similarly, follow-up on D28 may underestimate late treatment failures, particularly for drugs with a prolonged half-life. Our results corroborate those previously reported from various endemic countries [2-6,8-11,13-19]. These findings support the continued use of artemisinin-based combination treatments.

Of all the non-synonymous mutations, only the V589I mutation was associated with slower para-site clearance in patients receiving AL and ASSP treatment (r = 0.18). This differs from some studies conducted in Africa [2,4]. This discrepancy may be due to different methodological approaches. The A578S non-synonymous mutation, which was observed at a rate of 1.5% in Mali [2], was also reported in this study. However, these mutations are not among the validated or candidate resis-tance markers reported by the WHO [8,21]. These mutations reflect the genetic diversity of the *K13* gene and have been reported in Niger [5], Kenya [6], Guinea, the Democratic Republic of the Congo [15], and Senegal [10]. However, treatment failures with ASSP are due to mutations at codons 51, 59, and 108 of the *dhfr* gene and at codons 431, 436, and 581 of the *dhps* gene. These findings have been reported in previous studies [14]. The absence of a mutation at codon 540 supports the efficacy of sulfadoxine and, consequently, the efficacy of the ASSP combination.

## Conclusion

The study confirms the clinical and biological effi-cacy of ACTs in children in Boali in 2024. Analysis of the *K13* gene predominantly revealed wild-type alleles. The synonymous and non-synonymous mutations observed reflect the genetic diversity of the *K13* gene and are not associated with re-sistance to artemisinin-based drugs. To reduce disease transmission and prevent the emergence of resistance, it would be prudent to regularly monitor the efficacy of artemisinin-based drugs.

## Acknowledgments

We thank the participants for their coopera-tion during the study and the Boali Secondary Hospital staff for their support in ensuring the study’s success.

## Funding

This work was carried out with financial and technical support from the Global Fund and the World Health Organization (WHO).

## Authors’ contributions

DRA: data collection and laboratory analysis NMHB: review and revision of the manuscript NBR: data analysis

SP: data collection MZ: data collection

JDL: review and revision of the manuscript WSN: design, development, data analysis, val-idation of results, drafting, and submission of the manuscript

## Conflicts of interest

The authors declare no conflicts of interest.
